# Sedentary behavior patterns and adiposity in children: a study based on compositional data analysis

**DOI:** 10.1186/s12887-020-02036-6

**Published:** 2020-04-02

**Authors:** Aleš Gába, Željko Pedišić, Nikola Štefelová, Jan Dygrýn, Karel Hron, Dorothea Dumuid, Mark Tremblay

**Affiliations:** 1 Faculty of Physical Culture, Palacký University Olomouc, Olomouc, Czech Republic; 2 Institute for Health and Sport, Victoria University, Melbourne, Australia; 3 Faculty of Science, Palacký University Olomouc, Olomouc, Czech Republic; 4 Alliance for Research in Exercise, Nutrition and Activity, School of Health Sciences, University of South Australia, Adelaide, Australia; 5 Healthy Active Living and Obesity Research Group, Children’s Hospital of Eastern Ontario, Ottawa, Canada

**Keywords:** Accelerometry, Body mass index, Child behavior, Pediatric obesity, Sedentary behavior

## Abstract

**Background:**

Between-person differences in sedentary patterns should be considered to understand the role of sedentary behavior (SB) in the development of childhood obesity. This study took a novel approach based on compositional data analysis to examine associations between SB patterns and adiposity and investigate differences in adiposity associated with time reallocation between time spent in sedentary bouts of different duration and physical activity.

**Methods:**

An analysis of cross-sectional data was performed in 425 children aged 7–12 years (58% girls). Waking behaviors were assessed using ActiGraph GT3X accelerometer for seven consecutive days. Multi-frequency bioimpedance measurement was used to determine adiposity. Compositional regression models with robust estimators were used to analyze associations between sedentary patterns and adiposity markers. To examine differences in adiposity associated with time reallocation, we used the compositional isotemporal substitution model.

**Results:**

Significantly higher fat mass percentage (FM%; *β*_ilr1_ = 0.18; 95% CI: 0.01, 0.34; *p* = 0.040) and visceral adipose tissue (VAT; *β*_ilr1_ = 0.37; 95% CI: 0.03, 0.71; *p* = 0.034) were associated with time spent in middle sedentary bouts in duration of 10–29 min (relative to remaining behaviors). No significant associations were found for short (< 10 min) and long sedentary bouts (≥30 min). Substituting the time spent in total SB with moderate-to-vigorous physical activity (MVPA) was associated with a decrease in VAT. Substituting 1 h/week of the time spent in middle sedentary bouts with MVPA was associated with 2.9% (95% CI: 1.2, 4.6), 3.4% (95% CI: 1.2, 5.5), and 6.1% (95% CI: 2.9, 9.2) lower FM%, fat mass index, and VAT, respectively. Moreover, substituting 2 h/week of time spent in middle sedentary bouts with short sedentary bouts was associated with 3.5% (95% CI: 0.02, 6.9) lower FM%.

**Conclusions:**

Our findings suggest that adiposity status could be improved by increasing MVPA at the expense of time spent in middle sedentary bouts. Some benefits to adiposity may also be expected from replacing middle sedentary bouts with short sedentary bouts, that is, by taking standing or activity breaks more often. These findings may help design more effective interventions to prevent and control childhood obesity.

## Background

The reduction in global prevalence of obesity is one of the greatest public health challenges of today. The number of obese children and adolescents has increased dramatically in the past four decades [1]. According to recent worldwide estimates, 6% of girls and 8% of boys are obese [1]. Obese children are at an increased risk of several physical and psychological comorbidities in their childhood and chronic illness and premature death later in life [2, 3]. The origin of childhood obesity is complex, but the key contributing factor seems to be long-term dysregulation of energy balance. An excessive consumption of high-energy food contributes to disproportionally high energy intake, while the energy expenditure is too low as a result of an insufficient level of physical activity (PA). It was estimated that globally 81% of children and adolescents do not meet PA recommendations [4]. Many of them also spend most of their time being sedentary [5].

Sedentary behavior (SB) is defined as any waking behavior in a sitting, reclining or lying posture with an energy expenditure of ≤1.5 metabolic equivalent [6]. Excessive SB is considered a risk factor for several chronic diseases and conditions, including obesity [7, 8], and it has high prevalence, especially in developed countries [9]. SB is often assessed using hip-mounted accelerometers, where total SB is estimated as the amount of time with a low number of accelerometer counts, usually below the cut point of 100 counts per minute [10]. The risk of obesity appears to be affected not only by overall SB, but also by sedentary patterns, that is, by the way SB is accumulated [11]. Prolonged uninterrupted SB is considered to contribute to poor health, while interrupting SB with bouts of standing and PA may provide several health benefits [12]. Thus, understanding how patterns of SB accumulation are associated with health outcomes may have implications for interventions to prevent childhood obesity. While there is a relatively large evidence base on the associations between PA and adiposity and total SB and adiposity, much less is known about obesogenic effects of specific sedentary patterns.

Sedentary patterns can be expressed by sedentary bouts, that is, uninterrupted time spent in SB, and sedentary breaks, that is, the number of interruptions in SB. Children’s SB is considered to be highly fragmented because children accumulate high number of sedentary breaks during a day and spend most of their daily sedentary time in bouts of short duration, typically less than 10 min per bout [13–15]. To date, only a few studies have been conducted on the association between device-measured sedentary patterns and obesity markers in the pediatric population, and their findings are inconsistent [14, 16].

A systematic review by Cliff et al. [11] found that childhood obesity is more likely associated with the time spent in sedentary bouts than with the number of sedentary breaks. Similarly, Carson et al. [17] found a significant positive association between the time spent in sedentary bouts of short duration (< 10 min) and body mass index (BMI) in school-aged children. In this context, while analyzing the associations between sedentary patterns and obesity, it is necessary to consider the between-person differences in the time spent in sedentary bouts of different durations.

Previous research has also shown that the risk of obesity associated with excessive SB is more pronounced among those who are insufficiently physically active [17]. For this reason, analyses of the association between sedentary patterns and obesity should take into account individual PA levels. The amounts of time spent in SB and PA represent a sub-composition of the 24-h cycle [18, 19]. Given that the time in a day is constrained to 24 h, a change in the duration of one time-use component (e.g., time spent in PA) inevitably results in a change in the duration of one or more of the remaining time-use components (e.g., time spent in SB) [20]. In this context, in order to assess the association between SB and health, it is recommended to use a statistical approach that includes SB and PA variables in the same model [18, 20–22]. The use of compositional data analysis (CoDA) has been recommended over traditional multivariable models as it respects the compositional properties of the data by representing them as log ratios [22]. Moreover, this approach allows for the use a compositional version of isotemporal substitution analysis [23], to estimate a theoretical change in a health outcome resulting from a change in the duration of one type of behavior in favor of one or more remaining behaviors [24]. To our knowledge, no study has been published using the CoDA approach to analyze the association between sedentary patterns and adiposity markers in children. Therefore, the aim of the present study was to apply CoDA to investigate cross-sectional associations between adiposity and: (1) sedentary patterns, (2) reallocations of time spent in different sedentary bouts to PA, and (3) reallocations of time among sedentary bouts of different durations.

## Methods

### Participants

We used baseline data from a longitudinal study conducted between 2013 and 2019 among students in selected elementary schools in Moravia region, Czech Republic. The sample included schools from towns and cities of various population sizes. Sports academies and schools/classes with students with special needs were not included in the sample. Of the 24 elementary schools invited to participate in the study, one-third joined the study.

The sample included 632 children aged 7 to 12 years of age, who had no apparent medical problems that could affect their movement behaviors. The individuals who reported deliberate weight loss during the 12 months prior to the measurement period were excluded. The present analysis included children who had complete data for all variables of interest (*n* = 425; 58% girls).

### Sedentary behaviors assessment

SB and PA were assessed for seven consecutive days using a hip-mounted ActiGraph GT3X accelerometer (ActiGraph, LLC., FL, USA). A detailed description of the measurement protocol is provided elsewhere [25]. Briefly, the time sampling interval was set at 60s, non-wear time was defined using the Troiano algorithm [26], and the Evenson cut-off points [27] were used to estimate the time spent in SB, light PA (LPA), and moderate-to-vigorous PA (MVPA). A sedentary bout was defined as 1 or more consecutive minutes in which the accelerometer registered less than 100 counts per minute on the vertical axis. Sedentary patterns were expressed through the duration and frequency of sedentary bouts. We analyzed sedentary bouts of 1–9 min (short bouts), 10–29 min (middle bouts) and ≥30 min duration (long bouts). Accelerometer data were considered acceptable for analysis, if the participant wore the device for at least 4 days including 1 weekend day with ≥10 h of wear time per day.

### Adiposity assessment

Adiposity was expressed as fat mass percentage (FM%), fat mass index (FMI), and visceral adipose tissue (VAT), and was assessed by means of a multi-frequency bioelectrical impedance device (InBody 720 device; Biospace Co., Ltd., Seoul, Korea). Such assessment of body adiposity is considered sufficiently valid in the pediatric population [28]. During the measurement, the participants were in a standing position while barefoot and wearing light clothing. The participants were required to maintain adequate hydration for at least 24 h and fast for at least 4 h before the measurement. An experienced researcher conducted the measurement during the morning hours on school premises.

### Statistical analysis

The analyses were conducted in R software, version 3.4.2 (R Foundation for Statistical Computing, Vienna, Austria) using the *robCompositions* package and in the IBM Statistical Package for the Social Sciences (SPSS) software, version 23 (SPSS Inc., an IBM Company, Chicago, IL, USA). The means and standard deviations were calculated for the outcome measures. For compositional variables, the robust compositional means were calculated [19].

The CoDA approach was applied to assess the association between SB and adiposity. For the purpose of compositional regression analysis, compositions were mapped into real space using isometric log-ratio (*ilr*) transformation [29]. Specifically, compositional covariates were expressed through pivot coordinates which enable for interpretation in terms of dominance of a given compositional part with reference to the rest of components in the first coordinate (e.g., *ilr*_1_) [30]. For this purpose, the compositional parts were permutated, as explained in detail in previous papers [19, 22]. Linear models with robust estimators were used to eliminate the influence of possible outliers [19] that may occur in movement behavior data.

For each participant, two average waking-time compositions were generated. In Model 1, the *ilr*s of the three-part composition (SB, LPA and MPVA) were included as explanatory variables, while Model 2 was based on a 5-part composition of time spent in short, middle and long sedentary bouts, LPA, and MVPA. Regression models were adjusted for sex and age. Regression coefficient estimates corresponding to the first pivot coordinate (containing all the relative information about one particular compositional part) were of interest. Since few zero values occurred in long sedentary bouts (7 out of 425 cases), these were replaced by the two-thirds of the minimum non-zero value in that variable [31]. All dependent variables were log-transformed to honor the additive scale assumption and also to better accommodate the common model assumptions.

Regression estimates were used to predict the differences in adiposity status associated with the reallocation of time between different movement behaviors. Given the fact that the long sedentary bouts occurred only a few times during the week in the vast majority of participants, we decided to linearly adjust the robust mean composition to the theoretical sum of weekly waking hours, at an assumed 16 waking hours per day. This was based on the findings by Spruyt et al. [32] who have found that school-aged children usually sleep 8 h per day. We estimated differences in adiposity associated with reallocations of time between parts using the mean composition as a starting point. We calculated 95% confidence intervals (CIs) for the compositional isotemporal substitution estimates. Differences in adiposity were considered significant when 95% CIs did not cover zero. A compressive description of robust CoDA is available in a previous paper [19] and in Supplementary files.

## Results

Anthropometric, SB and PA characteristics stratified by sex are shown in Table 1. In the study sample, a total of 25.9% of children were overweight or obese. The prevalence of obesity was higher in boys (12.8%) than in girls (5.3%). Compared with boys, girls had lower average BMI *z*-score and higher FM% by 0.3 units (*p* = 0.001) and 1.5% points (*p* = 0.047), respectively.

**Table 1:** Descriptive characteristics and sedentary patters of study sample

	Girls (***N*** = 246)		Boys (***N*** = 179)		Total sample (***N*** = 425)	
	Mean	SD	Mean	SD	Mean	SD
Age (years)	9.8	1.3	9.9	1.2	9.8	1.3
Body height (cm)	142.3	8.8	143.8	9.4	142.9	9.1
Body weight (kg)*	35.4	8.1	37.8	9.7	36.4	8.9
BMI *z*-score*	0.2	1.1	0.5	1.2	0.3	1.1
Fat mass (kg)	7.0	4.2	7.0	5.4	7.0	4.7
Fat mass (%)*	18.4	7.4	16.9	8.3	17.8	7.8
Fat mas index (kg/m^2^)	3.4	1.9	3.3	2.3	3.3	2.1
Visceral adipose tissue (cm^2^)	32.1	22.9	36.4	28.5	33.9	25.5
Count per minute*	569.9	160.7	606.6	167.7	585.4	164.5
Wear time (h/day)	12.6	0.9	12.7	0.9	12.6	0.9
SB (min/day)	359.1	67.4	362.9	68.9	360.7	68.0
LPA (min/day)	348.0	52.6	339.2	55.7	344.3	54.0
MVPA (min/day)*	48.8	18.6	58.4	22.9	52.8	21.0
**Sedentary bouts analysis**						
Short sedentary bouts (min/day)*	202.0	27.9	192.1	27.1	197.8	28.0
Middle sedentary bouts (min/day)	114.3	39.3	120.5	42.7	116.9	40.8
Long sedentary bouts (min/day)*	42.8	33.2	50.3	33.2	46.0	33.4
Short sedentary bouts (number/day)*	79.1	10.7	73.7	10.3	76.8	10.8
Middle sedentary bouts (number/day)	7.5	2.4	7.8	2.6	7.6	2.5
Long sedentary bouts (number/day)*	1.1	0.8	1.2	0.8	1.1	0.8

*BMI* Body mass index, *LPA* Light intensity physical activity, *MVPA* Moderate-to-vigorous physical activity, *SB* Sedentary behavior, *SD* Standard deviation

*Significant difference between sexes, *t*-test (*p* < 0.05)

Short bout: 1–9 min, Middle bout: 10–29 min, Long bout: ≥30 min

The children spent 87% of their total SB time in sedentary bouts that were shorter than 30 min. Although no significant difference in the time spent in total SB was observed between sexes, boys spent on average 9.9 min/day (*p* < 0.001) less in short sedentary bouts and 7.5 min/day more in long sedentary bouts (*p* = 0.022) compared with girls. This corresponds with the difference in the number of short sedentary bouts per day; boys had on average 5.4 short sedentary bouts per day more than girls (*p* < 0.001). The median number of valid days of accelerometry was 6 and the mean wear time was 12.6 ± 0.9 h/day. No significant difference was observed between sexes in the number of valid days of accelerometry or wear time.

**Fig. 1:**
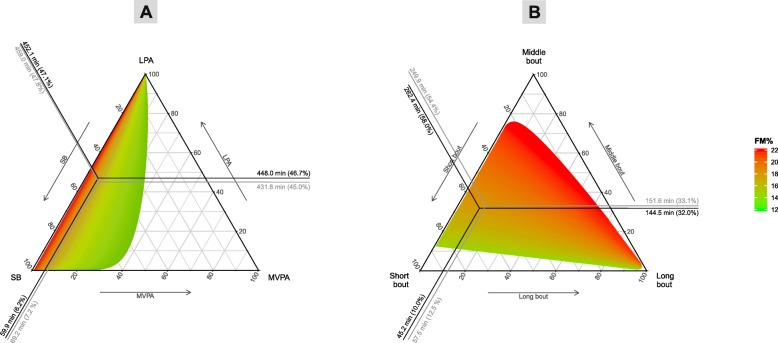
Ternary plots with predicted response in FM% for the composition of (**a**) waking hours and (**b**) total SB decomposed to bouts. FM% – fat mass percentage, LPA – light intensity physical activity, MVPA – moderate-to-vigorous physical activity, SB – sedentary behaviors. *Note*. Robust compositional mean was adjusted to 16 h of wake time

The ternary graphs presented in Fig. 1 (Panel A) and Figure S1 show the association of wake time behaviors with adiposity. The plots show that there was no change in any of the adiposity markers associated with the change in proportion of time spent in total SB (relative to remaining behaviors). The respective associations were also found by the regression analysis (Table 2, Model 1). The compositional isotemporal substitution analysis found significant associations only for VAT, even when 1 h/week from total SB was reallocated to MVPA. The isotemporal substitution analysis did not confirm significant differences in FM% and FMI when substituting time spent in total SB with LPA and MVPA (Tables 3 and 4).

**Table 2:** Compositional robust regression model estimates for the adiposity markers in total sample

	Fat mass (%)			Fat mass index (kg/m^**2**^)			Visceral adipose tissue (cm^**2**^)		
	*β*_ilr1_	(95% CI)	*p*-value	*β*_ilr1_	(95% CI)	*p*-value	*β*_ilr1_	(95% CI)	*p*-value
**Model 1**									
SB (h/week)	0.06	(−0.12, 0.24)	0.527	0.04	(−0.19, 0.27)	0.724	0.09	(−0.26, 0.44)	0.614
LPA (h/week)	0.13	(−0.08, 0.34)	0.230	0.18	(−0.09, 0.45)	0.200	0.32	(−0.11, 0.75)	0.140
MVPA (h/week)	**−0.19**	**(−0.32, −0.06)**	**0.005**	**−0.22**	**(−0.39, −0.05)**	**0.011**	**−0.41**	**(−0.67, −0.15)**	**0.002**
**Model 2**									
Short sedentary bouts (h/week)	−0.28	(−0.64, 0.08)	0.122	− 0.38	(−0.83, 0.08)	0.104	−0.55	(−1.30, 0.20)	0.148
Middle sedentary bouts (h/week)	**0.18**	**(0.01, 0.34)**	**0.040**	0.20	(−0.01, 0.41)	0.065	**0.37**	**(0.03, 0.71)**	**0.034**
Long sedentary bouts (h/week)	−0.02	(−0.08, 0.05)	0.629	−0.02	(−0.10, 0.07)	0.713	−0.07	(−0.20, 0.07)	0.332
LPA (h/week)	0.31	(0.00, 0.62)	0.053	0.41	(0.01, 0.80)	0.043	0.64	(0.00, 1.28)	0.050
MVPA (h/week)	**−0.18**	**(−0.30, −0.06)**	**0.003**	**−0.22**	**(−0.37, −0.06)**	**0.007**	**−0.39**	**(−0.63, −0.15)**	**0.001**

*BMI* Body mass index, *CI* Confidence interval, *ilr1* Isometric log-ratio (first coordinate), *LPA* Light intensity physical activity, *MVPA* Moderate-to-vigorous physical activity, *SB* Sedentary behavior

All dependent variables were transformed before analysis using the natural logarithm

Independent variables are expressed as the first pivot coordinate which represents the relative contribution of one behavior relative to remaining behaviors

All models were adjusted for sex and age

Bold values denote significant chance in adiposity status

**Table 3:** Estimated percentage change in adiposity markers associated with isotemporal substitutions between sedentary behavior and light-intensity physical activity

	Reallocation from SB to LPA				Reallocation from LPA to SB			
	1 h/week		2 h/week		1 h/week		2 h/week	
	Percentage change	(95% CI)	Percentage change	(95% CI)	Percentage change	(95% CI)	Percentage change	(95% CI)
**Fat mass (%)**								
Total SB	−0.2	(−0.7, 0.3)	−0.3	(−1.4, 0.8)	0.2	(−0.3, 0.7)	0.3	(−0.8, 1.4)
Short sedentary bouts	1.4	(−0.2, 3.0)	2.8	(−0.4, 6.2)	−1.4	(−2.9, 0.2)	−2.7	(−5.6, 0.4)
Middle sedentary bouts	−0.4	(−1.3, 0.4)	−0.9	(−2.6, 0.9)	0.4	(−0.5, 1.2)	0.6	(−0.9, 2.3)
Long sedentary bouts	0.8	(−0.5, 2.1)	−1.7	(−1.1, 4.6)	−0.8	(−1.9, 0.4)	−1.5	(−3.6, 0.6)
**Fat mas index (kg/m**^**2**^**)**								
Total SB	−0.1	(−0.8, 0.7)	−0.2	(−1.6, 1.2)	0.1	(−0.7, 0.8)	0.2	(−1.2, 1.6)
Short sedentary bouts	1.9	(−0.2, 3.9)	3.8	(−0.3, 8.0)	−1.8	(−3.7, 0.2)	−3.5	(−7.2, 0.3)
Middle sedentary bouts	−0.4	(−1.5, 0.7)	−0.8	(−3.0, 1.4)	0.3	(−0.7, 1.4)	0.5	(−1.5, 2.6)
Long sedentary bouts	1.0	(−0.7, 2.6)	2.0	(−1.6, 5.7)	−0.9	(−2.3, 0.5)	−1.9	(−4.5, 0.8)
**Visceral adipose tissue (cm**^**2**^**)**								
Total SB	0.2	(−1.0, 1.4)	0.3	(−1.9, 2.5)	−0.2	(−1.4, 1.0)	−0.3	(−2.5, 1.9)
Short sedentary bouts	2.8	(−0.5, 6.2)	5.7	(−1.0, 12.9)	−2.7	(−5.8, 0.5)	−5.3	(−11.2, 1.0)
Middle sedentary bouts	−0.9	(−2.6, 0.8)	−1.9	(−5.3, 1.6)	0.8	(−0.9, 2.4)	1.4	(−1.8, 4.7)
Long sedentary bouts	2.3	(−0.4, 4.9)	4.8	(−1.0, 10.9)	−2.0	(−4.3, 0.2)	−3.9	(−8.0, 0.3)

*CI* Confidence interval, *LPA* Light intensity physical activity, *SB* Sedentary behavior

**Table 4:** Estimated percentage change in adiposity markers associated with isotemporal substitutions between sedentary behavior and moderate-to-vigorous physical activity

	Reallocation from SB to MVPA				Reallocation from MVPA to SB			
	1 h/week		2 h/week		1 h/week		2 h/week	
	Percentage change	(95% CI)	Percentage change	(95% CI)	Percentage change	(95% CI)	Percentage change	(95% CI)
**Fat mass (%)**								
Total SB	−1.5	(−3.3, 0.3)	−2.8	(−6.2, 0.6)	1.7	(−0.4, 3.8)	3.6	(−1.1, 8.3)
Short sedentary bouts	−1.2	(−2.7, 0.4)	−2.0	(−5.1, 1.1)	1.5	(−0.3, 3.3)	3.4	(−0.3, 7.2)
Middle sedentary bouts	**−2.9**	**(−4.6, −1.2)**	**−5.6**	**(−8.7, −2.4)**	**3.2**	**(1.3, 5.2)**	**6.9**	**(2.8, 11.2)**
Long sedentary bouts	−1.7	(−3.4, 0.0)	−3.1	(−6.5, 0.4)	**2.1**	**(0.3, 3.9)**	**4.6**	**(0.8, 8.6)**
**Fat mas index (kg/m**^**2**^**)**								
Total SB	−2.2	(−4.5, 0.1)	−4.2	(−8.7, 0.3)	2.6	(−0.2, 5.4)	5.6	(−0.4, 11.6)
Short sedentary bouts	−1.2	(−3.3, 0.8)	−2.2	(−6.0, 1.8)	1.6	(−0.6, 3.9)	3.8	(−1.0, 8.8)
Middle sedentary bouts	**−3.4**	**(−5.5, −1.2)**	**−6.5**	**(−10.4, −2.4)**	**3.8**	**(1.4, 6.3)**	**8.2**	**(2.9, 13.7)**
Long sedentary bouts	−2.1	(−4.3, 0.2)	−3.8	(−8.2, 0.8)	**2.5**	**(0.2, 4.9)**	**5.6**	**(0.6, 10.9)**
**Visceral adipose tissue (cm**^**2**^**)**								
Total SB	**−6.4**	**(−9.8, −3.0)**	**−11.9**	**(−17.7, −5.6)**	**7.8**	**(3.5, 12.4)**	**17.7**	**(7.7, 27.7)**
Short sedentary bouts	−2.6	(−5.8, 0.6)	−4.7	(−10.6, 1.7)	3.4	(−0.2, 7.1)	7.8	(0.1, 16.1)
Middle sedentary bouts	**−6.1**	**(−9.2, −2.9)**	**−11.5**	**(−17.2, −5.5)**	**7.0**	**(3.2, 11.0)**	**15.4**	**(6.9, 24.6)**
Long sedentary bouts	−3.1	(−6.5, 0.3)	−5.5	(−12.0, 1.5)	**4.1**	**(0.4, 7.9)**	**9.3**	**(1.5, 17.8)**

*CI* Confidence interval, *MVPA* Moderate-to-vigorous physical activity, *SB* Sedentary behavior

Bold values denote significant chance in adiposity status

**Fig. 2:**
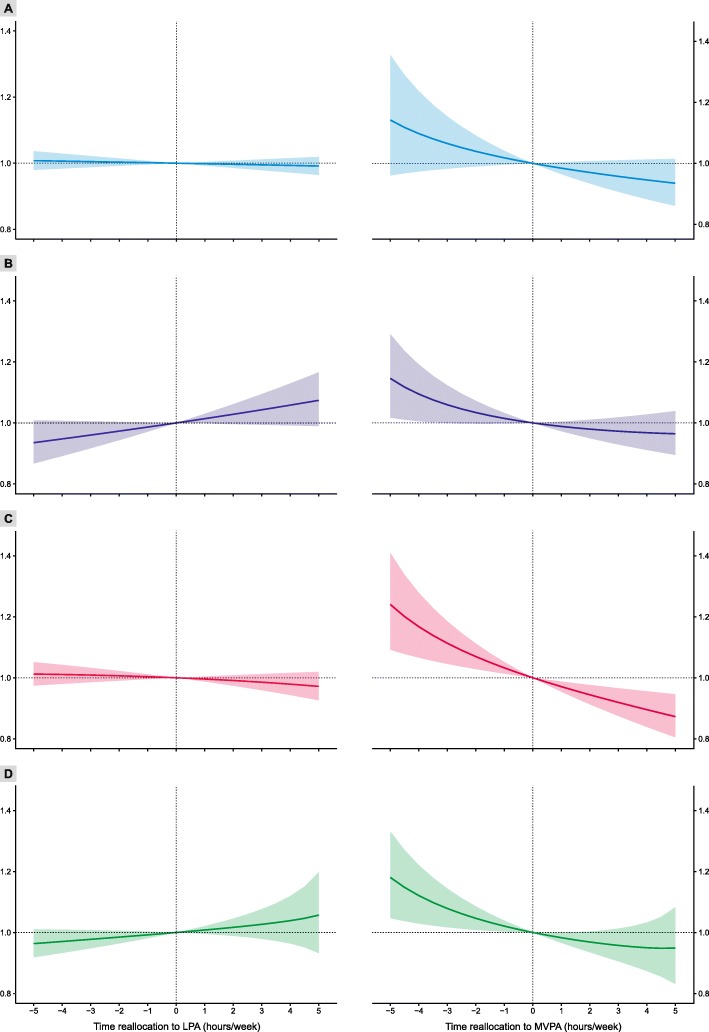
Estimated relative changes in FM% for reallocations of time between sedentary behavior (SB) and physical activity (PA). LPA – light-intensity physical activity, MVPA – moderate-to-vigorous physical activity. *Note*: (**a**) total SB, (**b**) short bouts of SB, (**c**) middle bouts of SB and (**d**) long bouts of SB

More time spent in middle sedentary bouts (relative to remaining behaviors) was associated with higher FM% (*β*_ilr1_ = 0.18; 95% CI: 0.01, 0.34; *p* = 0.040) and VAT (*β*_ilr1_ = 0.37; 95% CI: 0.03, 0.71; *p* = 0.034) (Table 2). Reallocation of time spent in middle sedentary bouts to MVPA was associated with a significantly lower FM% (Fig. 2, Panel C). For example, reallocation of 1 h/week from middle sedentary bouts to MVPA was associated with 2.9% (95% CI: 1.2, 4.6), 3.4% (95% CI: 1.2, 5.5), and 6.1% (95% CI: 2.9, 9.2) lower FM%, FMI and VAT, respectively (Tables 3 and 4). The associations of reallocating sedentary time to and from MVPA were asymmetric (Fig. 2).

The associations between the relative contributions of different sedentary bouts and FM% are displayed in Fig. 1 (Panel B). A higher proportion of time spent in middle sedentary bouts was associated with higher FM%. Although no significant changes in adiposity were associated with reallocating time from long to short sedentary bouts and vice versa, substituting time spent in middle sedentary bouts with short sedentary bouts was associated with favorable adiposity changes. For example, substituting 2 h/week of time spent in middle sedentary bouts with short sedentary bouts was associated with 3.5% (95% CI: 0.02, 6.9) lower FM% (Table S1 and Figure S2).

## Discussion

By using accelerometer estimates and a CoDA approach, we found that Czech school-aged children spend most of their waking time in SB and that their sedentary patterns are associated with their adiposity. Simulated reallocations of time from middle sedentary bouts to MVPA or to shorter sedentary bouts were associated with favorable adiposity changes.

SB showed high fragmentation, because a vast majority of all sedentary bouts consisted of short sedentary bouts. These results correspond with a study by Saunders et al. [14] who observed a similarly high number of short sedentary bouts in school-aged children. It is therefore obvious that, contrary to the adult population where short sedentary bouts account only for ~70% of all sedentary bouts [33], SB in children appears to be dominantly of an intermittent nature. This may be explained by age-related changes in sedentary patterns that occur during the transition from childhood to adolescence and later to adulthood. Increasing age leads to a decrease in fragmentation of SB, and the largest changes occur between 9 and 12 years of age [13].

This study did not find significant associations between total SB (relative to remaining waking behaviors) and adiposity markers, however reallocations of time from total SB to MVPA were associated with lower VAT. Specifically, our findings suggest that breaking up sedentary bouts of 10–29 min (i.e., middle sedentary bouts) might be a useful strategy in the prevention and control of childhood obesity. Our sample size was sufficiently large to ensure statistical power of the regression models of 0.55–0.63, if the population effect size was small (i.e., *f*^2^ = 0.02, according to Cohen [34]). It may therefore be that non-significant results for short and long sedentary bouts were obtained simply because the population effect sizes were small. This will have to be confirmed in future studies, using larger samples. It is possible, however, that sedentary bouts in duration of less than 10 min are interrupted with enough episodes of PA to avoid negative effects on adiposity status. It may also be that long sedentary bouts are interrupted with longer periods of PA than medium sedentary bouts. Future research should explore these plausible patterns of SB and PA, as this was beyond the scope of the current study.

School-related SB accounts for approximately 44% of the total daily SB [35] and compared with before and after school time it typically has a higher number of middle sedentary bouts [15]. Although the present study did not include an analysis of sedentary patterns in different domains and segments of the day, it could be assumed that many middle sedentary bouts were accumulated in the school setting. School-based interventions might, therefore, be particularly useful for reducing the number and duration of middle sedentary bouts. Other intervention targets could be screen-time and passive transportation, both of which significantly contribute to total SB [36] and potentially result in the accumulation of middle sedentary bouts.

Although previous studies found significant effects of interrupting SB with brief bouts of LPA among adolescents [37], among younger elementary school children we observed a significant association with adiposity only for reallocation of sedentary time in favor of MVPA. This might suggest that the benefits of LPA in relation to adiposity status are greater among adolescents than among children. Another way to reduce adiposity may be to facilitate a change in fragmentation of SB, instead of focusing on MVPA. We found that substituting middle sedentary bouts with short sedentary bouts was associated with a more favorable adiposity status. Such a change in fragmentation of SB would result in an increase of the number of episodes of quiet standing or PA but may not necessarily affect their total duration. This finding supports the significance of SB interventions that are based on postural changes. It should be noted that expected effects of changes in fragmentation of SB are lower than the ones associated with reallocation of SB in favor of MVPA. This is also supported by recent findings of an experimental study by Betts et al. [38], who observed only a 12% change in energy expenditure between sitting and standing, which per se may not be sufficient for the treatment of obesity.

Our findings suggest that the association between SB and adiposity is asymmetric, that is, dependent on whether the time was reallocated to or from SB. For example, whereas no significant decrease in adiposity was found when the time spent in long sedentary bouts was reallocated to MVPA, a significant increase in adiposity was found even for the reallocation of only 1 h/week of MVPA to long sedentary bouts. Declining levels of PA and increasing SB can be observed as children grow older, especially in those with higher BMI [39]. The present results highlight not only the necessity for obesity interventions to reduce SB, but also to prevent the age-related decrease in PA in favor of SB. Future studies should explore these relationships in adolescents, as the present study only included children up to 12 years of age. In addition to an asymmetric response in adiposity, different responses in adiposity depending on the distribution of body fat were observed. Although a significant association was observed between reallocation of time spent in middle sedentary bouts and total adiposity (represented by FM%), a two-fold greater relationship with VAT was observed in our sample. Moreover, a significant response to reallocation of total SB to MVPA was observed only in the case of VAT.

### Strengths and limitations

A strength of this study was that we used the CoDA approach, which adequately deals with the compositional properties of time-use data. Although in recent years several studies used the CoDA approach to assess the associations between device-measured SB and obesity markers [19, 40–44], to our knowledge there has been no CoDA-based study analyzing the effect of sedentary patterns on adiposity in children.

There are also several limitations that should be considered when interpreting the results of this study. First, its cross-sectional design enabled us to provide only theoretical estimates of the isotemporal substitution effects. The findings should be interpreted with caution and confirmed by longitudinal and intervention studies. Second, the analyses did not include sleep duration, which is an important component of the 24-h cycle. There is evidence on the association of insufficient sleep duration and obesity [45], and sleep recommendations were, therefore, incorporated in several national [46–48] and World Health Organization 24-h movement guidelines [49]. Third, the results of compositional regression analysis should be interpreted consistent with the compositional approach. The regression estimates correspond to multivariate, log-transformed data (i.e., pivot coordinates) and cannot be interpreted in a univariate sense. Interpretation is multivariate, that is, the estimates correspond to the change in one component, relative to the change in the remaining components of waking-time composition.

Last, there are some factors that may affect the estimates of the association between SB and adiposity which we could not account for in this study. For example, eating patterns and total energy consumption are associated with movement behaviors and childhood obesity [50–52]. However, a recently published CoDA-based study found that waking-time movement behaviors are associated with several adiposity markers independent of unhealthy eating patterns [44]. Other potential limitations include accelerometer data collecting and processing. The sampling interval (i.e., epoch length) or accelerometer data processing (e.g., choice of accelerometer cut-off point) could affect the estimated amount of time spent in PA, total SB and different bouts of SB [53]. Total SB could also include time spend in standing because we were not able to differentiate between sitting and idle standing. We took into consideration only acceleration on the vertical axis when SB was analyzed. This approach does not also allow capture of so-called dynamic sitting [54] which has higher energy expenditure than quite sitting and may potentially affect the adiposity status of children. It should be also noted that compositional data was linearly adjusted to an expected mean amount of time (i.e., 16 waking hours per day). In this context, estimated differences in adiposity associated with time reallocation may vary between children with different total waking time. The use of predictive techniques for the assessment of VAT may have influenced the results of the analysis.

## Conclusions

In conclusion, reallocation of time to MVPA from middle sedentary bouts seems to be associated with the most favorable adiposity markers among children. Beneficial associations were also observed for reallocating time from middle sedentary bouts to short sedentary bouts. An improvement in adiposity status can be expected even when 1 h/week from middle sedentary bouts is reallocated to MVPA. Moreover, changes in in fragmentation of SB were also associated with favorable adiposity markers. These findings may help inform more effective interventions to prevent and control childhood obesity.

## Supplementary information

**Additional file 1: Figure S1.** Ternary plots with predicted response in FMI and VAT for composition of waking hours. FMI – fat mass index, LPA – light intensity physical activity, MVPA – moderate-to-vigorous physical activity, SB – sedentary behaviors, VAT – visceral adipose tissue. *Note*. Robust compositional mean was adjusted to 16 h of wake time.

**Additional file 2: Figure S2.** Estimated relative changes in FM% for reallocationsof time between sedentary bouts. FM% – fat mass percentage, LPA – light intensity physical activity, MVPA – moderate-to-vigorous physical activity.

**Additional file 3.** Detailed description of compositional data analysis.

**Additional file 4: Table S1.** Estimated percentage change in adiposity markers associated with reallocations of time between sedentary bouts.

## Data Availability

The dataset analyzed during the current study is available in the Figshare repository, 10.6084/m9.figshare.11980068.

## Abbreviations

BMI: Body mass index
CoDA: Compositional data analysis
FMI: Fat mass index
FM%: Fat mass percentage
LPA: Light physical activity
MVPA: Moderate-to-vigorous physical activity
PA: Physical activity
SB: Sedentary behavior
VAT: Visceral adipose tissue
