# Supplementary figures and images for: Sedentary behavior patterns and adiposity in children: a study based on compositional data analysis
Source: BMC Pediatr. 2020 Apr 2;20:147. doi: 10.1186/s12887-020-02036-6 (PMC7114780; doi:10.1186/s12887-020-02036-6)

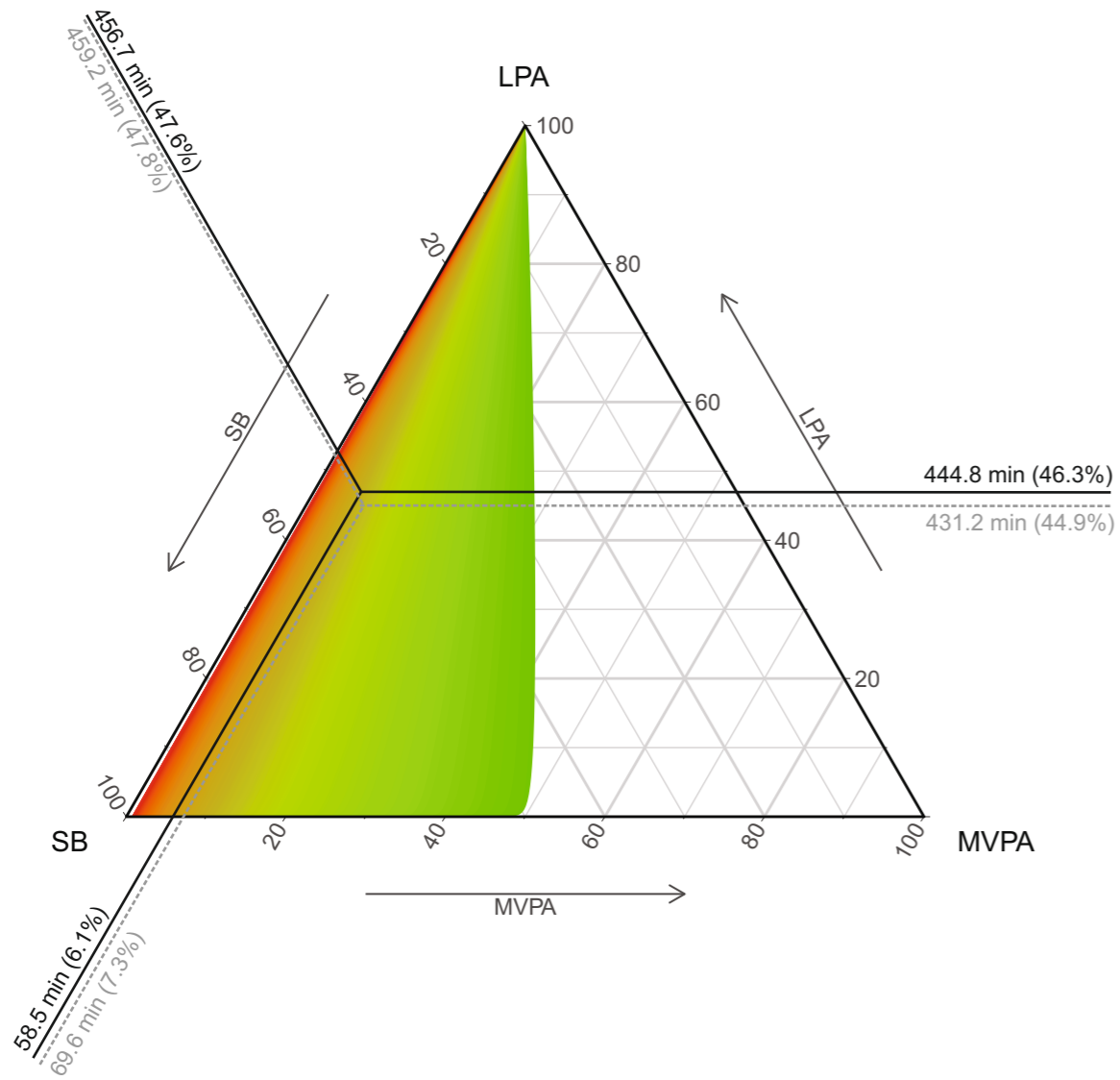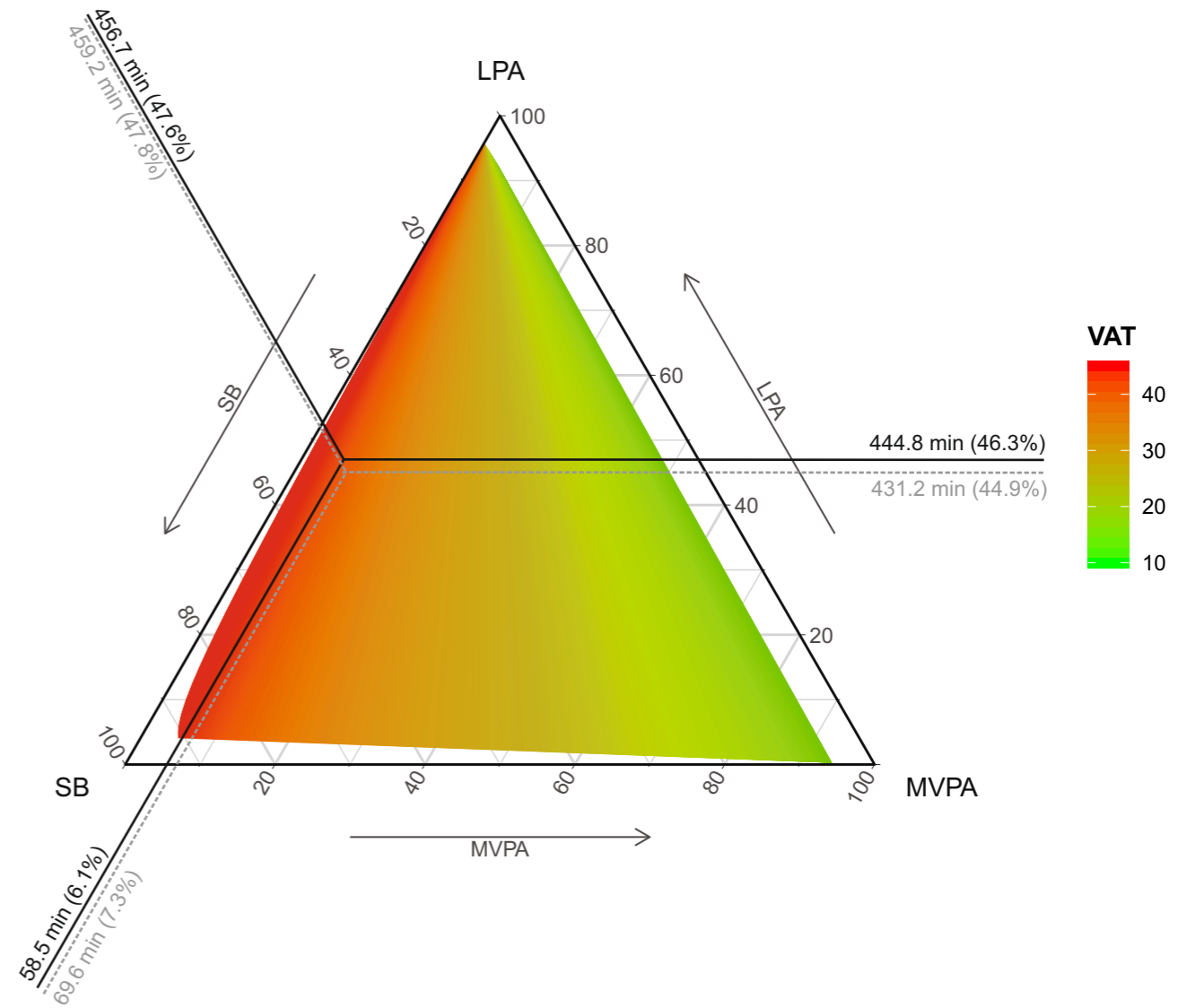

Supplement: Supplementary file 1 — Additional file 1: Figure S1. Ternary plots with predicted response in FMI and VAT for composition of waking hours. FMI – fat mass index, LPA – light intensity physical activity, MVPA – moderate-to-vigorous physical activity, SB – sedentary behaviors, VAT – visceral adipose tissue. Note. Robust compositional mean was adjusted to 16 h of wake time. [file 12887_2020_2036_MOESM1_ESM.pdf]

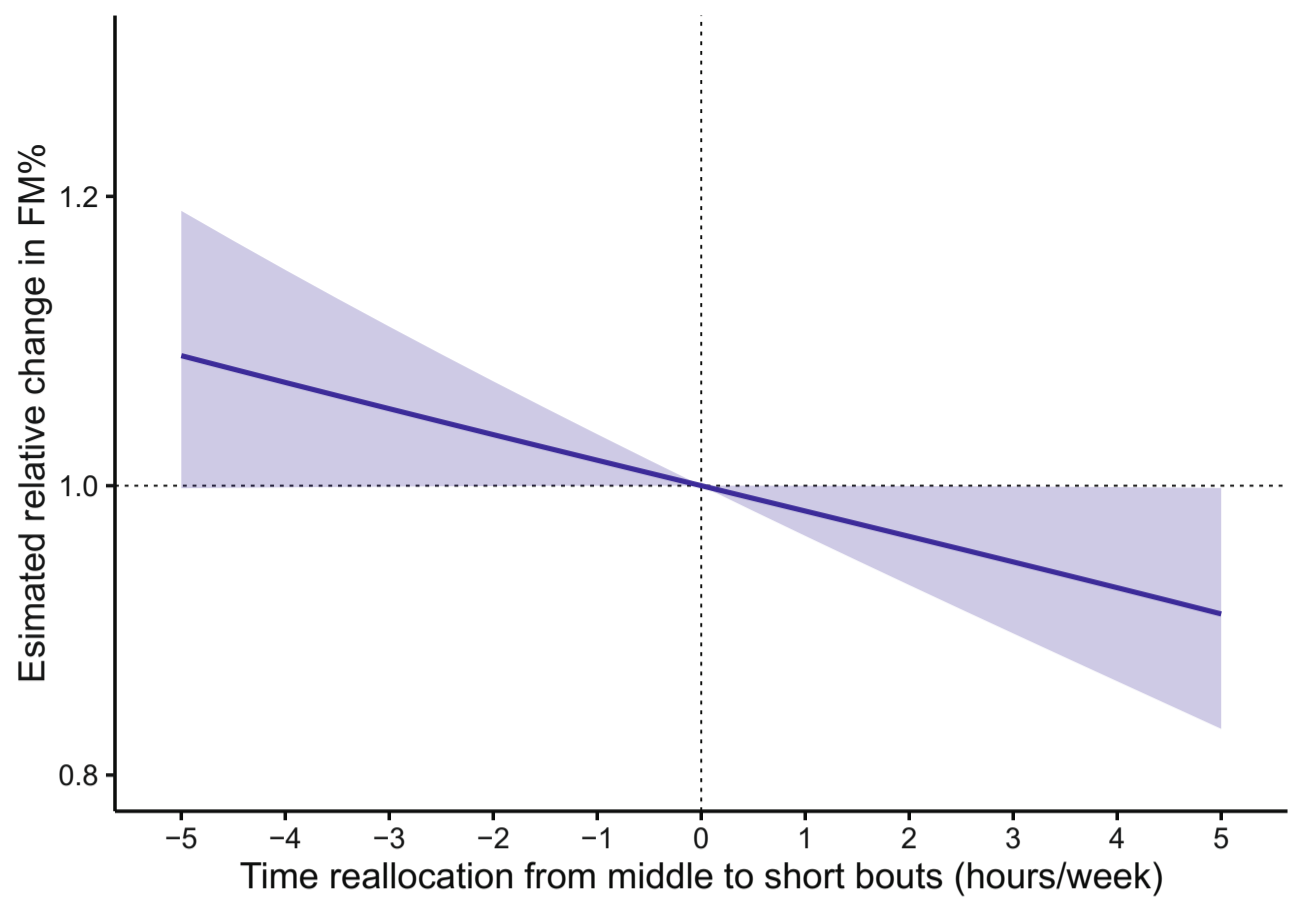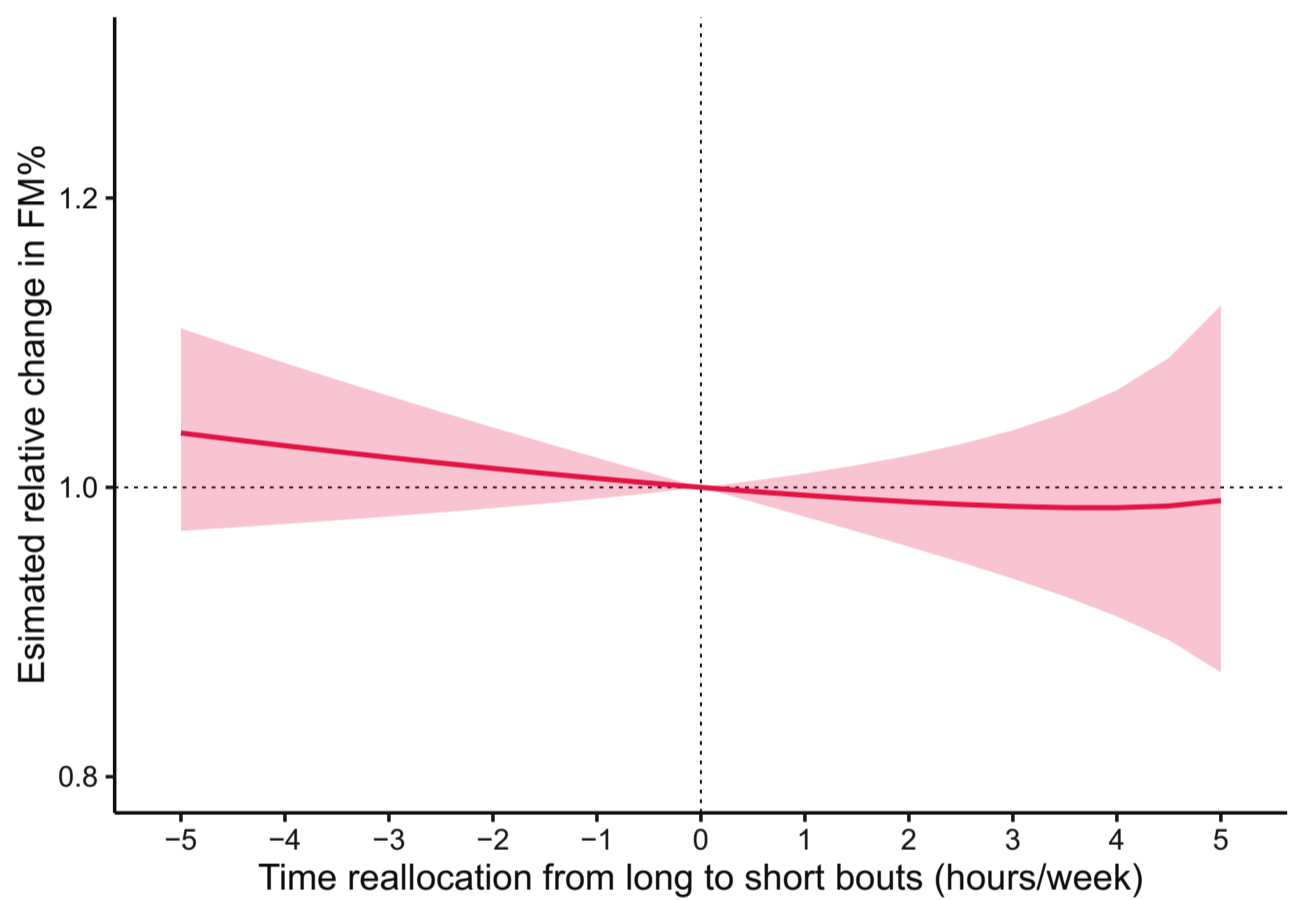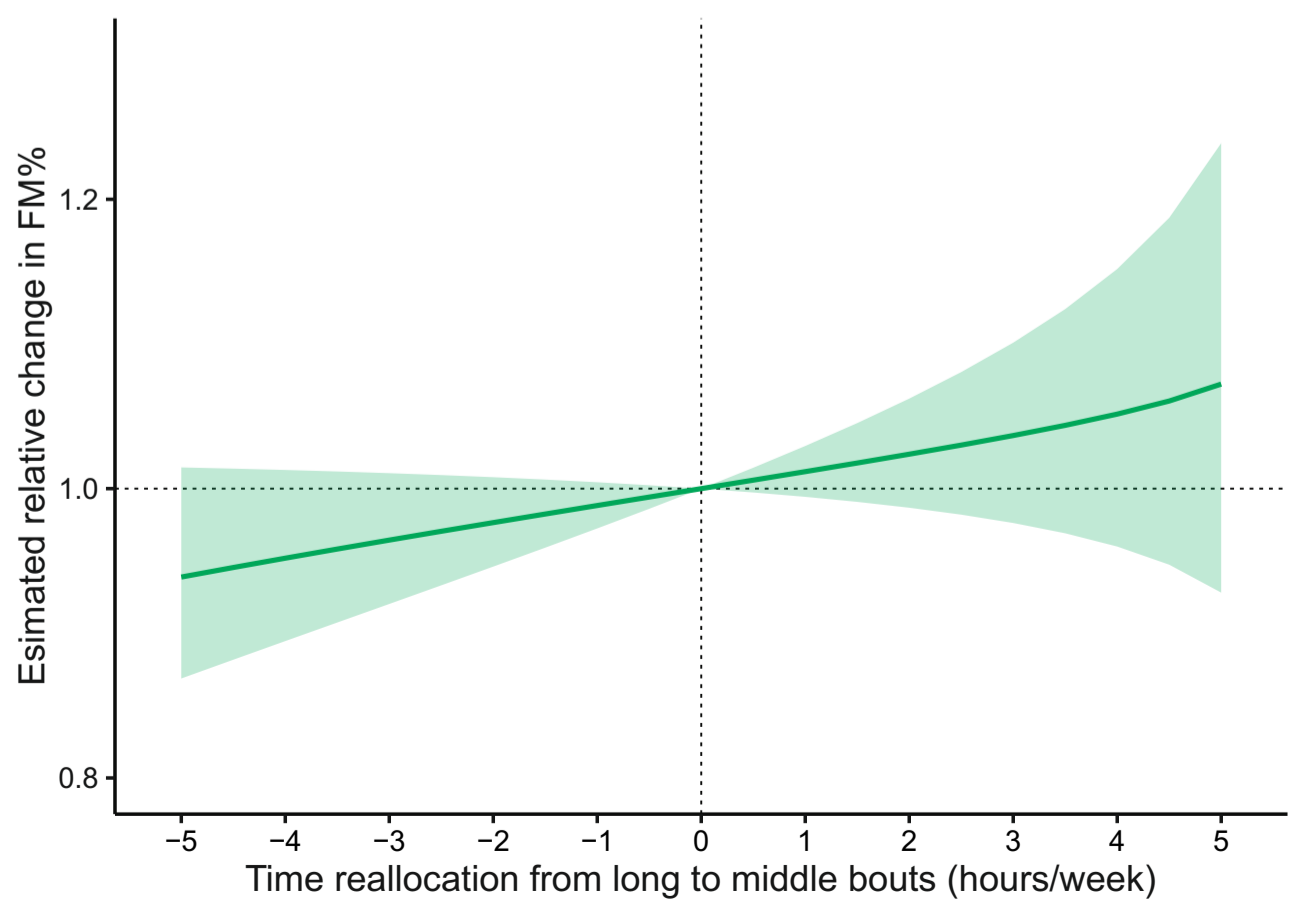

Supplement: Supplementary file 2 — Additional file 2: Figure S2. Estimated relative changes in FM% for reallocationsof time between sedentary bouts. FM% – fat mass percentage, LPA – light intensity physical activity, MVPA – moderate-to-vigorous physical activity. [file 12887_2020_2036_MOESM2_ESM.pdf]
