# Supplementary material for: Sedentary behavior patterns and adiposity in children: a study based on compositional data analysis
Source: BMC Pediatr. 2020 Apr 2;20:147. doi: 10.1186/s12887-020-02036-6 (PMC7114780; doi:10.1186/s12887-020-02036-6)
Supplement: Supplementary file 3 — Additional file 3. Detailed description of compositional data analysis. [file 12887_2020_2036_MOESM3_ESM.docx]

## Detailed description of compositional data analysis

To describe the statistical analysis in more detail, lets denote the movement behavior composition as $\boldsymbol{x}=(x_{1},\ldots, x_{D})'$. The corresponding pivot coordinates $\boldsymbol{z}=(z_{1},\ldots, z_{D-1})'$ are constructed as $z_{j}=\sqrt{\frac{D-j}{D-j+1}.}\ln\frac{x_{j}}{\sqrt[D-j]{\prod_{k=j+1}^{D} x_{k}}}, j=1, \ldots, D-1.$ The following regression model is considered

$\ln Adiposity=\beta_{0}+\beta_{1}z_{1}+\ldots+\beta_{D-1}z_{D-1}+\beta_{D}Age+\beta_{D+1}Gender+\varepsilon$,

where $\boldsymbol{\beta}=(\beta_{0}, \ldots, \beta_{D+1})'$ is a vector of unknown regression coefficients and $\varepsilon$ is the error of model. Actually, $D$ different regression models are considered (here $D=3$ and $D=5$, respectively) with $D$ different sets of pivot coordinates – each time, different part is put at the first position in a composition so that each first pivot coordinate represents the relative dominance of a different compositional part. Let $\bar{\boldsymbol{z}}$ denotes the (robust) mean composition expressed in pivot coordinates and let asterisk mark the connection with the mean composition where some amount of time was reallocated between the two parts. Then ${\bar{\boldsymbol{z}}}^{\boldsymbol{*}}$ stands for the reallocated mean composition expressed in pivot coordinates. The difference between the predicted adiposity markers (on the log scale) for the mean composition and the predicted adiposity markers (on the log scale) for the reallocated mean composition is computed as $\hat{\ln Adiposity-}\ln\hat{Adiposity}^{*}=\boldsymbol{c}'\hat{\boldsymbol{\beta}}$, where $c=(0,$ $\bar{\boldsymbol{z}}\boldsymbol{-}{\bar{\boldsymbol{z}}}^{\boldsymbol{*}}\boldsymbol{,}0,0\boldsymbol{)'}$ and $\hat{\boldsymbol{\beta}}$ is a vector of estimated regression coefficients (calculated using the robust MM-regression). The corresponding approximate confidence interval is calculated as $\left\langle\boldsymbol{c}^{\boldsymbol{'}}\hat{\boldsymbol{\beta}}-t_{n-k}(1-\alpha/2)\sqrt{\boldsymbol{c}'\hat{\mathrm{var} \hat{\boldsymbol{\beta}}}\boldsymbol{c,}} \boldsymbol{c}^{\boldsymbol{'}}\hat{\boldsymbol{\beta}}+t_{n-k}(1-\alpha/2)\sqrt{\boldsymbol{c}'\hat{\mathrm{var} \hat{\boldsymbol{\beta}}}\boldsymbol{c}} \right\rangle$, where $t_{n-k}(1-\alpha/{2)}$ is a $(1-\alpha/{2)}$-quantile of *t*-distribution with $n-k$ degrees of freedom, $n$ is a number of observations, $k$ is a number of regression parameters (here $k=D+2$) and $\hat{\mathrm{var} \hat{\boldsymbol{\beta}}}$ is estimated covariance matrix of the regression coefficients; the quantiles hold only approximately in the robust case. Then, the estimated difference in adiposity associated with the reallocation of time between movement behaviors is considered as significant if the confidence interval does not include 0. Subsequently, the relative difference between the predicted adiposity markers for the mean composition and the predicted adiposity markers for the reallocated mean composition is computed as $\hat{Adiposity}/{\hat{Adiposity}^{*}}=\exp\boldsymbol{(c}'\hat{\boldsymbol{\beta}})$. Similarly, for the confidence interval, the exponential function is applied to its lower and upper value. Then, the significance is implied if the confidence interval does not include 1.
