# Supplementary material for: Sedentary behavior patterns and adiposity in children: a study based on compositional data analysis
Source: BMC Pediatr. 2020 Apr 2;20:147. doi: 10.1186/s12887-020-02036-6 (PMC7114780; doi:10.1186/s12887-020-02036-6)
Supplement: Supplementary file 4 — Additional file 4: Table S1. Estimated percentage change in adiposity markers associated with reallocations of time between sedentary bouts. [file 12887_2020_2036_MOESM4_ESM.docx]

**Table S1**. Estimated percentage change in adiposity markers associated with reallocations of time between sedentary bouts.

|  | |  | **Reallocation to short sedentary bouts** | | | | |  | **Reallocation from short sedentary bouts** | | | | |
| --- | --- | --- | --- | --- | --- | --- | --- | --- | --- | --- | --- | --- | --- |
|  |  |  | **1 h/week** | |  | **2 h/week** | |  | **1 h/week** | |  | **2 h/week** | |
|  |  |  | Percentage  change | (95% CI) |  | Percentage  change | (95% CI) |  | Percentage  change | (95% CI) |  | Percentage  change | (95% CI) |
| **Fat mass (%)** | |  |  |  |  |  |  |  |  |  |  |  |  |
|  | Middle sedentary bouts |  | –1.8 | (–3.5, 0.0) |  | **–3.5** | **(–6.9, –0.02)** |  | 1.8 | (–0.01, 3.6) |  | 3.5 | (–0.03, 7.2) |
|  | Long sedentary bouts |  | –0.5 | (–2.0, 1.0) |  | –1.0 | (–4.1, 2.2) |  | 0.6 | (–0.8, 2.0) |  | 1.3 | (–1.4, 4.1) |
|  |  |  |  |  |  |  |  |  |  |  |  |  |  |
| **Fat mas index (kg/m^2^)** | |  |  |  |  |  |  |  |  |  |  |  |  |
|  | Middle sedentary bouts |  | –2.2 | (–4.3, 0.1) |  | –4.3 | (–8.5, 0.1) |  | 2.2 | (–0.1, 4.5) |  | 4.4 | (–0.1, 9.1) |
|  | Long sedentary bouts |  | –0.8 | (–2.7, 1.1) |  | –1.5 | (–5.4, 2.5) |  | 0.9 | (–0.9, 2.7) |  | 1.9 | (–1.6, 5.5) |
|  |  |  |  |  |  |  |  |  |  |  |  |  |  |
| **Visceral adipose tissue (cm^2^)** | |  |  |  |  |  |  |  |  |  |  |  |  |
|  | Middle sedentary bouts |  | –3.5 | (–7.0, 0.1) |  | –7.0 | (–13.6, 0.1) |  | 3.6 | (–0.1, 7.4) |  | 7.3 | (–0.2, 15.4) |
|  | Long sedentary bouts |  | –0.5 | (–3.4, 2.5) |  | –0.6 | (–6.7, 5.8) |  | 0.7 | (–2.1, 3.6) |  | 1.7 | (–3.8, 7.4) |
| CI – confidence interval.  Bold values denote significant chance in adiposity status. | | | | | | | | | | | | | |
